# An OTU deubiquitinating enzyme in *Eimeria tenella* interacts with *Eimeria tenella* virus RDRP

**DOI:** 10.1186/s13071-018-2626-x

**Published:** 2018-01-31

**Authors:** Pu Wang, Jianhua Li, Pengtao Gong, Weirong Wang, Yongxing Ai, Xichen Zhang

**Affiliations:** 1 College of Veterinary Medicine, Jilin University, Changchun, 130062 China; 2 College of Animal Science, Jilin University, Changchun, 130062 China

**Keywords:** *Eimeria tenella*, Etv-RDRP, Et-OTU, Interaction, Deubiquitinase, Mutation, Enhanced

## Abstract

**Background:**

Chicken coccidiosis, a disease caused by seven species of *Eimeria* (Apicomplexa: Coccidia), inflicts severe economic losses on the poultry industry. *Eimeria tenella* is the one of the most virulent species pathogenic to chickens. Many parasitic protozoans are parasitised by double-stranded (ds) RNA viruses, and the influence of protozoan viruses on parasitic protozoans has been extensively reported. *E. tenella* RNA virus 1 (Etv) was identified in *E. tenella*, and the complete genome sequence of Etv was analysed. Here, we screened Etv-RNA-dependent RNA polymerase (RDRP)-interacting host protein *E. tenella* ovarian tumour (OTU) protein-like cysteine protease (Et-OTU) using a yeast two-hybrid system with pGBKT7-RDRP plasmid serving as bait. A previous study demonstrated that Et-OTU could regulate the telomerase activity of *E. tenella*, indicating that Et-OTU affects *E. tenella* proliferation. However, whether Etv-RDRP affects the molecular biological characteristics of *E. tenella* by interacting with OTU remains unclear.

**Results:**

We obtained seven positive clones from the initial screen, and six of the seven preys were identified as false-positives. Finally, we identified an RDRP-associated protein predicted to be an *E. tenella* OTU protein. A α-galactosidase assay showed that the bait vector did not activate the GAL4 reporter gene, indicating no autoactivation activity from the RDRP bait fusion. Pull-down and co-immunoprecipitation assays verified the interaction between Et-OTU and Etv-RDRP both intracellularly and extracellularly. Additionally, Et-OTU was able to deconjugate K48- and K6-linked di-ubiquitin (di-Ub) chains *in vitro* but not K63-, K11-, K29-, or K33-linked di-Ub chains. The C239A and H351A mutations eliminated the deubiquitinase (DUB) activity of Et-OTU, whereas the D236A mutation did not. Additionally, when combined with RDRP, the DUB activity of Et-OTU towards K48- and K6-linked chains was significantly enhanced.

**Conclusion:**

Etv-RDRP interacts with Et-OTU both intracellularly and extracellularly. Etv-RDRP enhances the hydrolysis of Et-OTU to K6- or K48-linked ubiquitin chains. This study lays the foundation for further research on the relationship between *E. tenella* and Etv.

## Background

Chicken coccidiosis is induced by seven species of *Eimeria* (Apicomplexa: Coccidia) and can cause intestinal bleeding and death in chickens, threatening the health and welfare of poultry and causing serious economic losses in the poultry industry worldwide [1, 2]. Prevention and control of coccidiosis mostly rely on live vaccines and anticoccidial drugs. Considering drug resistance and pathogeny spreading, there are no effective methods for prevention and control of coccidiosis in chickens [3, 4] mainly because the cellular and molecular biological characteristics of chicken coccidia are unclear. Seven species are pathogenic to chickens, and *Eimeria tenella* is considered one of the most virulent [5].

*Trichomonas vaginalis*, *Leishmania aethiopica*, *Giardia duodenalis*, *Eimeria stiedae*, *E. brunetti* [6] and *E. necatrix* [7] are parasitised by double-stranded (ds) RNA viruses, and the influence of protozoan viruses on parasitic protozoans has been extensively reported. *Trichomonas vaginalis* virus can reduce the infectivity of *T. vaginalis*, and the presence of *Leishmania* RNA virus (LRV) in *Leishmania guyanensis* parasites contributes to exacerbation of the disease [8]. The high multiplicity of infection (MOI) of *Giardia duodenalis* induces growth arrest and reduces the vitality of *Giardia* [9, 10]*.* The dsRNA virus *E. tenella* virus (Etv) was identified in *E. tenella*, and the Etv genome was sequenced and analysed [11, 12]. Compared with other protozoan viruses, *Eimeria* viruses are more closely related to mycoviruses (fungal viruses), indicating a different evolutionary branch. Mycovirus infections in *Helminthosporium victoriae* and mitovirus infections in *Botrytis cinerea* can reduce the hypovirulence or virulence of their host phytopathogenic fungi and may, therefore, be employed as potential drug targets and biological control agents for plant fungal diseases [13, 14]. However, whether the presence of Etv affects the molecular biological characteristics of *E. tenella* remains unclear.

The complete genome sequence of Etv was recently published, and Etv encodes an RNA-dependent RNA polymerase (Etv-RDRP). In some viruses, RDRP exhibits RNA-dependent RNA polymerase activity, which is responsible for the transcription of genes encoding enzymes that function in both replication and transcription [15, 16]. Many RDRP-interacting proteins have been identified in humans, plants, and other eukaryotes. Protein kinase C-related kinase 2 (PRK2), a Ser/Thr kinase in the cAMP-dependent, cGMP-dependent and protein kinase C (AGC) kinase subfamily, binds and phosphorylates hepatitis C virus (HCV) RDRP, which has a positive regulatory role in HCV RNA replication [17]. Cellular Ser/Thr protein phosphatase 6 (PP6) interacts with and positively regulates the activity of influenza virus RDRP [18]. Although the effects of RDRP-interacting proteins on RDRP activity and viral replication are common, the regulatory effect of RDRP on host proteins is rarely reported.

In the current study, to study the effects of Etv-RDRP on the biological characteristics of *E. tenella*, we searched for proteins interacting with Etv-RDRP. We used a yeast two-hybrid system to screen Etv-RDRP-associated proteins using an *E. tenella* cDNA library as bait and identified an *E. tenella* ovarian tumour (OTU) protein-like cysteine protease (Et-OTU). Further investigation revealed that Etv-RDRP interacts with this Et-OTU and enhances the deubiquitinating activity of Et-OTU. In our previous study, down-regulation of Et-OTU expression levels decreased the telomerase activity of *E. tenella* [19]. Telomerase can maintain telomere length, promote cell proliferation and prolong cell life [20]. Thus, our results indicated that Etv might regulate *E. tenella* proliferation and ageing through the effect of Etv-RDPR on Et-OTU.

## Methods

### Plasmid construction

To construct RDRP bait plasmid used for yeast two-hybrid screening, a 550–1899 bp sequence of RDRP gene of Etv (GenBank: YP_009115500.1) (wild-type (WT) Etv was isolated from Changchun, China) was PCR amplified from cDNA of purified *E. tenella* and cloned into pGBKT7 between EcoRI and BamHI sites. Similarly, *rdrp* gene of Etv was also cloned into pGADT7 vector. Full-length prey genes (OTU-like cysteine protease, hypothetical protein, ribosomal protein L22, and uncharacterized ancient conserved region (ACR)) were amplified from *E. tenella* cDNA and cloned into pGBKT7 vector.

The full-length positive prey (Pp) plasmid sequence was cloned between BamHI and XhoI sites of pFast-Bac™ dual vector (ThermoFisher Scientific, Waltham, USA) fused to a glutathione S-transferase (GST) tag following the pH promoter. Etv-RDRP gene was ligated into XhoI and HindIII sites of pFast-Bac™HTA, generating a hexa-tagged protein.

### Yeast two-hybrid screening

For the yeast two-hybrid screen, a 550–1899 bp sequence of the Etv RDRP gene (GenBank: YP_009115500.1), which encodes the finger, palm and thumb domains, was cloned into pGBKT7 and used as bait. Using a Matchmaker Library Construction and Screening Kit (Clontech, Mountain View, USA), we constructed an *E. tenella* cDNA library using pGADT7 vector as prey. The yeast strain Y187 was transformed with the bait plasmid pGBKT7-Etv-RDRP, and a α-galactosidase assay was performed to examine autoactivation. The yeast strain Y187 was transformed with the preys, which contained a GAL4 activation domain. For interaction mating, the library and bait strains were co-cultured at 30 °C for 20 h. After mating, the culture was plated on selection media lacking histidine, leucine, tryptophan and adenine and containing 20 μg/ml X-α-gal (SD/-Ade/-His/-Leu/-Trp/X-α-gal). The resultant blue colonies were selected for further analysis. Co-cultured pGBKT7-53 and pGADT7-T plasmids were used as positive control, and co-cultured pGBKT7-Lam and pGADT7-T plasmids were used as negative controls. To reconfirm the hits from the above screen, Pp plasmid cloned into pGBKT7 from the initial screen was co-transformed into Y187 strain with the bait plasmid pGADT7-Etv-RDRP and plated on selection medium.

### Pull-down assays

The interaction between Etv-RDRP and Pp protein was further confirmed using pull-down assays. Purified Pp protein and glutathione-Sepharose 4B beads (GE Healthcare, Little Chalfont, UK) were incubated at 4 °C for 2 h in lysis buffer (10 mM Tris-HCl, pH 7.5, 150 mM NaCl, 1 mM EDTA, NP-40 and a complete protease inhibitor cocktail from Roche (Basel, Switzerland)). Next, the column was extensively washed with phosphate-buffered saline (PBS). Hexa-His-tagged Etv-RDRP protein was then added to Pp protein-bound resin, followed by mixing in lysis buffer at 4 °C for 4 h. Next, the resin mixture was extensively washed with PBS to remove unbound proteins, and the beads were eluted with sodium dodecyl sulfate-polyacrylamide gel electrophoresis (SDS-PAGE) loading buffer and boiled. Eluted proteins were separated on a 4–12% SDS-PAGE gel. Western blotting (WB) was performed for the identification of proteins of interest using a primary antibody against mouse hexa-His tag (TransGen Biotech, Beijing, China) at 1:1000 and a horseradish peroxidase (HRP)-conjugated goat anti-mouse IgG secondary antibody (TransGen Biotech) at 1:3000.

### Co-immunoprecipitation and western blot analysis

The pFast-Bac™pH-Pp and pFast-Bac™HTA-Etv-RDRP plasmids were transfected into competent DH_10_Bac bacteria to construct the respective recombinant bacmids. The recombinant bacmids were then extracted (Axygen, San Francisco, CA, USA) and transfected into Sf9 cells using FuGENE HD transfection reagent (Roche). Sf9 cells were maintained in Sf-900™II medium (Gibco, Carlsbad, CA, USA), and recombinant baculoviruses were harvested from transfected cells incubated at 27 °C every 7 days. The titres of third-generation viruses were determined using a Baculovirus Titering Kit (Expression Systems, Las Vegas, NV, USA), and third-generation viruses with titres over 5 × 10^7^ pfu/ml were used to infect Sf9 cells at an MOI of 1 for protein expression.

Sf9 cells were co-infected with the third-generation viruses of two recombinant baculovirus strains harbouring GST-Pp or Etv-RDRP. After 48 h, the infected cells were harvested and then lysed with precooled lysis buffer (50 mM Tris-HCl (pH 7.4), 150 mM NaCl, 5 mM EDTA, 1% NP-40, 5% glycerol and a protease inhibitor cocktail from Roche) for 30 min on ice. Next, the cell lysates were precleared with protein A-agarose (Millipore, Billerica, Massachusetts) at 4 °C for 30 min. The supernatants were then incubated with rabbit polyclonal antibodies against GST tag (Cell Signaling Technology (CST), Boston, MA, USA) at 4 °C overnight, and the resultant immune complexes were mixed with protein A-conjugated Sepharose beads at 4 °C for 2 h. Protein A-conjugated bead-bound immune complexes were then centrifuged for 5 s at 8000× *g* at 4 °C, and the supernatants were removed. The protein A beads were subsequently washed with wash buffer (50 mM Tris-HCl (pH 8.0), 500 mM NaCl, 5 mM EDTA and 1% NP40) three times to remove unbound proteins. The bound proteins were eluted with elution buffer (0.1 M glycine, pH 2.8), neutralised with neutralisation buffer (1 M Tris-HCl, pH 9.5), and separated via 12% SDS-PAGE. WB was performed to identify bound proteins using a primary rabbit polyclonal antibody against GST (CST) at 1:1000 and a primary mouse monoclonal antibody against hexa-His (Millipore) at 1:1000. Goat anti-rabbit IgG (1:3000, TransGen Biotech) and goat anti-mouse IgG (1:3000, TransGen Biotech) HRP secondary antibodies were then employed. Immunoprecipitation was performed with mouse monoclonal antibodies against hexa-His (Millipore) and protein A-agarose. The bound proteins were analysed via WB using a rabbit polyclonal antibody against GST (CST) at 1:1000 and a rabbit polyclonal antibody against the hexa-His tag (CST) at 1:1000. An HRP secondary antibody was employed with goat anti-rabbit IgG at 1:3000 (TransGen Biotech).

### Analysis of the Pp plasmid

The selected Pp plasmid was sequenced by Comate Bioscience Co. Ltd. (Jilin, Changchun, China) and analysed using BLASTX from the National Center for Biotechnology Information (NCBI) website. The conserved *Pp* gene sequence was aligned and compared with TgOTUD3A (*Toxoplasma** gondii*; GenBank: EPR62955.1), Otubain 2 (human; SW: Q96DC9), Otubain 1 (human; SW: Q96FW1), A20 (human; SW: P21580), Cezanne (human; SW: Q9NQ53) and VCIP135 (rat; SW: Q8CF97).

### Cloning, site-directed mutagenesis, expression, and purification of et-OTU in Sf9 cells

The full-length Et-OTU sequence (1107 bp open reading frame, GenBank: XM_013374305.1) was PCR-amplified from *E. tenella* cDNA using the primers OTU-F: 5′-ATG GTG CGC ACA TGT TTT GAC TC-3′ and OTU-R: 5′-CTA TCC CGG CTT ACT TGG CGT G-3′ and then cloned into pFast-HTA dual vector fused to a GST tag following the pH promoter. The catalytic core was predicted through alignment with other OTU proteins. The residues of the putative catalytic triad, comprising Cys, Asp and His, in the catalytic core were mutated to Ala (C239A, D236A and H351A) via site-directed mutagenesis using the primer pairs OCM-F: 5′-CAT CCG TAG GGG CCG GCA ACG CCC AG-3′ and OCM-R: 5′-CTG GGC GTT GCC GGC CCC TAC GGA TG-3′, ODM-F: 5′-CAT CCG TAG GGG CCG GCA ACT GCC AG-3′ and ODM-R 5′-CTG GCA GTT GCC GGC CCC TAC GGA TG-3′, and OHM-F: 5′-CGC CCA TAG CCT ACA ATG CCT TC-3′ and OHM-R: 5′-GAA GGC ATT GTA GGC TAT GGG CG-3′. Recombinant bacmids containing WT Et-OTU and the mutants Et-OTU (C239A), Et-OTU (D236A) and Et-OTU (H351A) were transfected into Sf9 cells to obtain the desired recombinant baculoviruses. Third-generation baculoviruses were used to infect Sf9 cells for protein expression. The cells were subsequently harvested and lysed in lysis buffer (10 mM Tris-HCl (pH 7.5), 150 mM NaCl, 1 mM EDTA, NP-40 and a complete protease inhibitor cocktail from Roche) via sonication following centrifugation at 8000× *g* for 15 min. Next, the supernatants, containing 5 mM dithiothreitol (DTT), were mixed with glutathione-Sepharose 4B at 4 °C for 1 h. The beads were then extensively washed to remove unbound proteins, and the bound proteins were eluted in elution buffer (20 mM reduced glutathione in 50 mM Tris-HCl, pH 8.0). The purified proteins were aliquoted and either used directly or stored at -80 °C.

### Characterisation of the et-OTU deubiquitination enzyme substrate preference *in vitro*

A linkage-specific deubiquitination assay was performed as follows: recombinant Et-OTU^WT^ or 10 μM Et-OTU^C239A^, 10 μM Et-OTU^D236A^ or 10 μM Et-OTU^H351A^ was diluted to 5 μM in deubiquitinase (DUB) dilution buffer (25 mM Tris, 150 mM NaCl and 10 mM DTT, pH 7.5) and incubated at room temperature for 15 min. Then, 10 μM solutions of K6-, K11-, K29-, K27-, K33-, K48-, or K63-linked di-ubiquitin (di-Ub) chains (R&D Systems, Minneapolis, USA) were diluted to a final concentration of 5 μM in 10 × DUB reaction buffer (500 mM Tris, 500 mM NaCl and 50 mM DTT, pH 7.5). The reactions were initiated by mixing 10 μl of enzyme stock and 10 μl of the di-Ub solution, followed by incubation at 37 °C for the indicated times. Reactions were stopped by the addition of SDS-PAGE loading buffer, and reaction mixtures were run on a 4–15% Tris-glycine gradient gel. The proteins were then transferred to a polyvinylidene difluoride (PVDF) membrane, and immunoblotting was performed with a primary antibody against ubiquitin (Ub; R&D Systems) at 1:2000 and a goat IgG anti-rabbit HRP secondary antibody (TransGen Biotech) at 1:3000.

### Effect of RDRP on the activity of OTU deubiquitination enzyme *in vitro*

The GST-OTU protein and GST-OTU-Hexa-tagged-RDRP protein complex were purified with glutathione-Sepharose 4B, and their concentrations were measured using a bicinchoninic acid (BCA) protein assay kit. GST-OTU and GST-OTU-Hexa-tagged-RDRP proteins were individually diluted to 5 μM in DUB dilution buffer and incubated at room temperature for 15 min. Then, 10 μM solutions of K6- and K48-linked di-Ub chains (R&D Systems) were individually diluted to a final concentration of 5 μM in 10× DUB reaction buffer. The reactions were initiated by mixing the enzyme stock and di-Ub solutions, followed by incubation at 37 °C for the indicated times. The reactions were stopped by the addition of 6× SDS loading buffer. The hydrolytic efficiencies of Et-OTU and Etv-RDRP combined with Et-OTU to K6 or K48 were compared.

## Results

### Screening Etv-RDRP-interacting host proteins

**Fig. 1:**
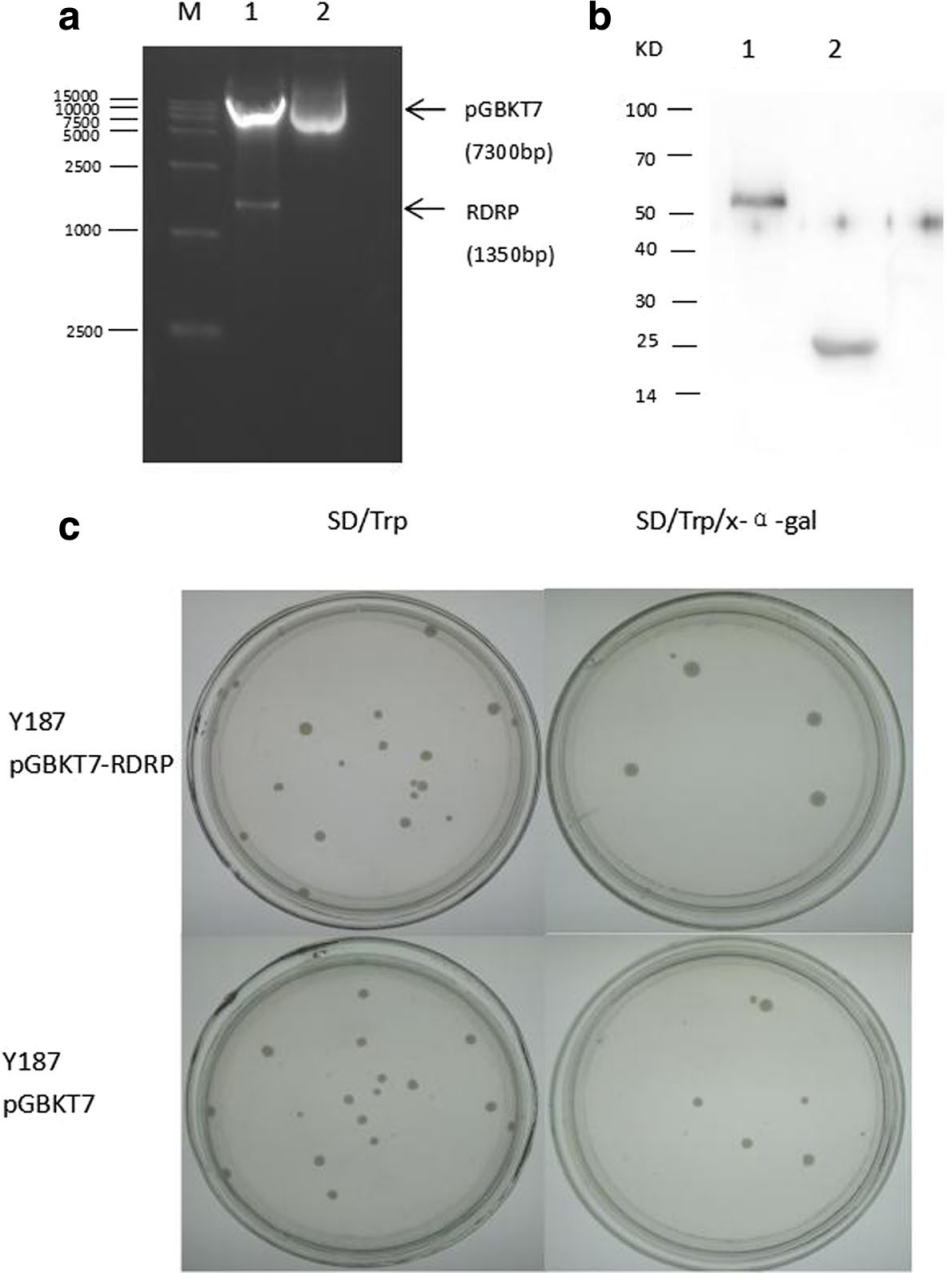
Construction of RDRP bait plasmids and testing of RDRP expression, toxicity, and autoactivation in yeast cells. **a** Agarose gel electrophoresis analysis of the bait plasmids pGBKT7-RDRP and pGBKT7. M: DNA marker; Lane 1: bait plasmid pGBKT7-RDRP digested with NdeI and SalI; Lane 2: pGBKT7 vector. **b** Immunoblot analysis of total proteins in the yeast strain Y187 transformed with the indicated plasmids. Lane 1: pGBKT7-RDRP; Lane 2: pGBKT7 vector. **c** Determination of the autoactivation and toxicity of RDRP bait in yeast cells. Yeast strain Y187 was transformed with pGBKT7-RDRP (upper) or pGBKT7 (lower) and grown on SD/-Trp and SD/-Trp/-X-α-gal plates. Lack of autoactivation is indicated by white colonies (or pink colonies) on SD/-Trp and SD/-Trp/-X-α-gal plates. Toxic bait is indicated by colonies significantly smaller than those containing the pGBKT7 empty vector

Digestion of pGBKT7-RDRP plasmid with *EcoR*I and *BamH*I resulted in two clear bands at the expected sizes (1350 bp for RDRP and 7.3 kb for pGBKT7 vector backbone) (Fig. 1a), suggesting successful cloning of RDRP. To evaluate the expression of the bait-fusion protein in yeast cells, we extracted total proteins from Y187 cells transformed with pGBKT7-RDRP via the urea/SDS method and analysed them via immunoblotting using an anti-Myc antibody. As shown in Fig. 1b, the RDRP bait fused to GAL4 DNA-binding domain was detected at the expected size of 53 kDa. Y187 containing the empty vector pGBKT7 expressing the 21 kDa GAL4 DNA-binding domain was used as the negative control. Before the yeast two-hybrid screen, the autoactivation activity of RDRP bait was tested. The bait plasmid pGBKT7-RDRP was transformed into the Y187 strain, and transformants were grown on SD/-Trp and SD/-Trp/x-α-gal plates. Autoactivation enables the expression of reporter genes and results in blue colonies grown on SD/-Trp and SD/-Trp/x-α-gal plates, but no autoactivation activity was detected from RDRP bait fusion (Fig. 1c). The normal growth of Y187 containing pGBKT7-RDRP (compared with the same strain harbouring pGBKT7 empty vector) on SD/-Trp plates indicated that there was no significant toxicity associated with RDRP bait-fusion protein in yeast cells (Fig. 1c). Collectively, these results suggested that the RDRP bait is suitable for yeast two-hybrid screening.

We obtained 7 positive clones from the initial screen. Y187 cells were co-transformed with pGBKT7 empty vector and each of the prey plasmids and grown on SD/-Trp/-Leu and SD/-Trp/-Leu/-His/-ADE/-x-α-gal plates. False-positive clones were indicated by blue colonies growing on both types of plates. Thus, 1 of the 7 prey proteins was identified as a false-positive according to the SD/-Trp/-Leu/-His/-ADE/-x-α-gal selection plates, and the remaining 6 were sequenced using a T7 promoter sequencing primer. BLAST searches showed that 1 of the 6 clones did not match any coding sequence (CDS), while 5 of the clones exhibited high sequence similarity to 4 known genes, encoding a hypothetical protein (AHB82174.1), putative ribosomal protein L22 (XP_013228850), putative uncharacterised ACR (CDJ42927) and putative OTU-like cysteine protease domain-containing protein (OTU, XP_013229759). Data for the prey proteins identified are presented in Table 1.

**Table 1:** BLAST results and putative functions of positive hits from the yeast two-hybrid screen

GenBank ID	Homologs in GenBank	Putative function
AHB82174.1	Hypothetical protein, protein, partial (mitochondrion)	Unknown
XP_013228850	ribosomal protein L22, putative	DNA repair and cell differentiation
CDJ42927	Uncharacterized ACR, YagE family COG1723 domain-containing protein, putative	Unknown
XP_013229759	OTU-like cysteine protease domain-containing protein, putative	Deubiquitinase

### An OTU-like protein was confirmed to interact with RDRP both intracellularly and extracellularly

**Fig. 2:**
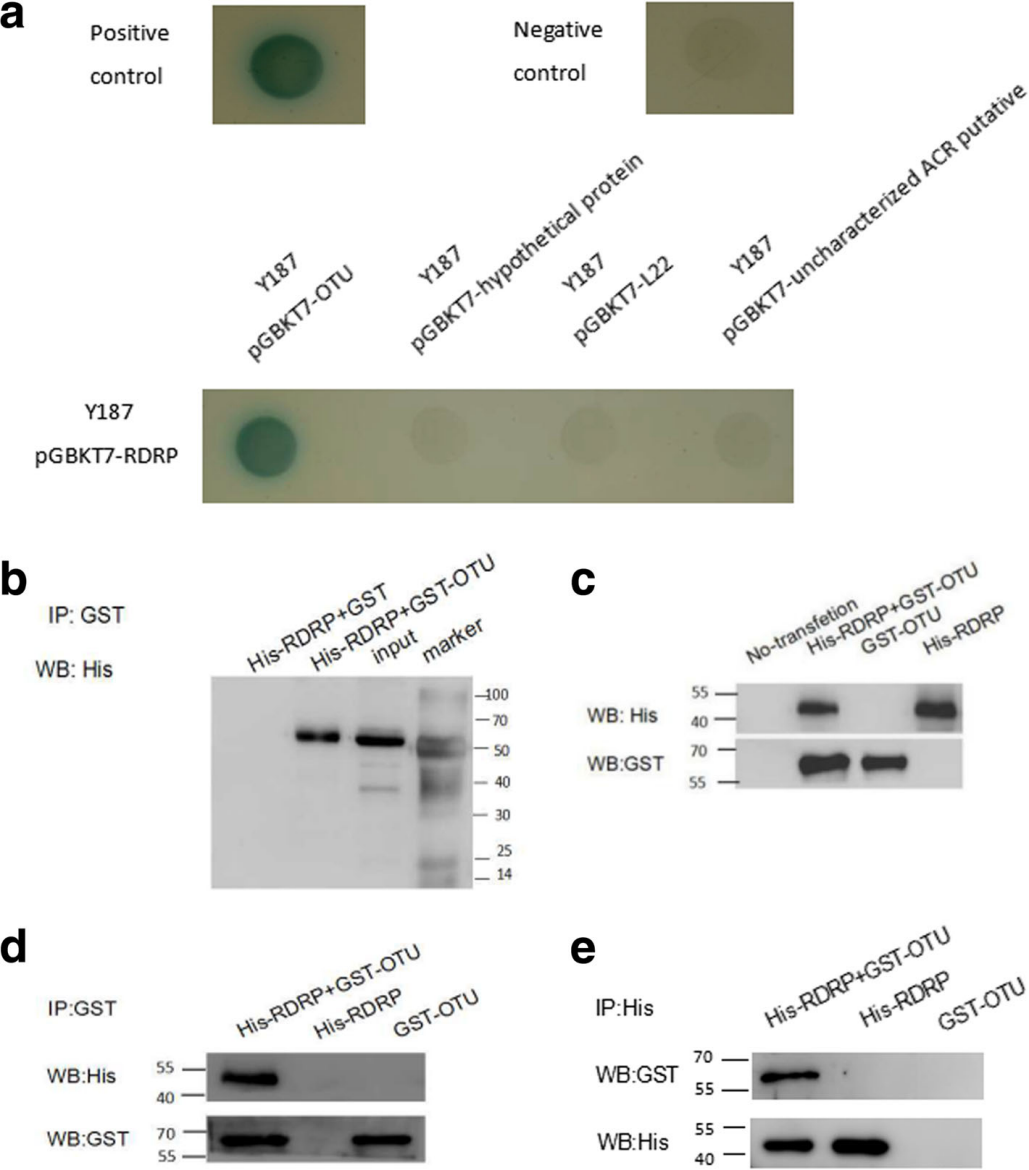
Confirmation of both intracellular and extracellular interactions between RDRP and host proteins. **a** Yeast two-hybrid testing of RDRP-host protein interactions. Yeast strain Y187 was co-transformed with pGADT7-RDRP and pGBKT7-prey and plated on SD/-Trp/-Leu/-His/-ADE/-x-α-gal plates. Positive interactions were indicated by the presence of blue colonies. The co-transformation of pGBKT7-53 and pGADT7-T was used as a positive control, whereas the co-transformation of pGBKT7-Lam and pGADT7-T was used as a negative control. **b** GST pull-down assay confirming extracellular interactions between RDRP and OTU. The binding of His-RDRP to GST-OTU immobilised on glutathione beads was determined by Western blot analysis. The total protein eluted from the beads was probed with an anti-His antibody as indicated. **c** The intracellular interaction between RDRP and host OTU proteins as determined by coimmunoprecipitation. DH10Bac bacteria were transfected with pFast-HTA-RDRP and pFast-dual-OTU to construct the corresponding recombinant bacmids. The recombinant bacmids were then extracted and transfected into Sf9 cells. The recombinant baculoviruses were incubated with transfected cells at 27 °C and harvested every 7 days. Third-generation viruses were employed to infect Sf9 cells for protein expression. After co-transfection of OTU and RDRP recombinant baculoviruses for 48 h, cell lysates were analysed by Western blotting with anti-His and anti-GST antibodies. Immunoprecipitation was performed with an anti-His antibody (**d**) and an anti-GST antibody (**e**) and detected by Western blotting with anti-His and anti-GST antibodies. Untransfected cells were used as controls

We obtained 4 predicted positive plasmids from the initial screen. To double-test the interactions between RDRP and the above-identified hits, a point-to-point yeast two-hybrid assay was performed. The full-length CDSs of the hypothetical protein, ribosomal protein L22, uncharacterised ACR and OTU-like cysteine protease domain-containing protein were amplified from *E. tenella* cDNA and cloned into pGBKT7 vector. The Y187 strain was transformed with pGBKT7 prey plasmids and pGADT7-RDRP, and the cultures were grown on selection and indication plates. After 4 days of growth at 30 °C, the cultures containing pGBKT7-OTU were able to grow on the selection plates and produce blue colonies, whereas those harbouring the ribosomal protein L22, uncharacterised ACR and hypothetical protein were not (Fig. 2a). The results showed that the last three sequences were probably false-positive hits in the yeast two-hybrid system, whereas the first OTU-like cysteine protease domain-containing protein is likely a true RDRP binding partner.

As shown in Fig. 2b, WB analysis indicated that the His-tagged Etv-RDRP protein bound directly to GST-Et-OTU fusion protein *in vitro* but not to GST alone under the same conditions, consistent with the results of the two-hybrid assay.

A Co-IP assay was performed using Sf9 cells expressing His-tagged RDRP and GST-tagged OTUs, and the precipitates of the lysed cells were analysed by WB (Fig. 2c). After incubation with anti-GST antibodies and protein A-agarose, GST-tagged OTU was co-precipitated with His-tagged RDRP (Fig. 2d). Subsequently, we conducted Co-IP with an anti-His antibody followed by WB with an anti-GST antibody, showing that Et-OTU bound Etv-RDRP but not the control (Fig. 2e). These results confirmed that OTU interacts with RDRP both intracellularly and extracellularly.

### The pp plasmid was predicted to encode an OTU protein

**Fig. 3:**
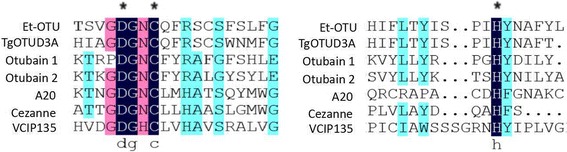
Conserved sequence alignment of catalytic Cys, Asp and His residues of the OTU family of DUBs. Amino acid alignment of TgOTUD3A (*Toxoplasma* *gondii*; GenBank: EPR62955.1), Otubain 2 (human; SW: Q96DC9), Otubain 1 (human; SW: Q96FW1), A20 (human; SW: P21580), Cezanne (human; SW: Q9NQ53) and VCIP135 (rat; SW: Q8CF97). The critical amino acid residues comprising the catalytic triad (Asp, Cys, and His) are highly conserved across these species (black asterisks) despite their evolutionary distance. The catalytic residues Asp, Cys, and His were mutated to generate Et-OTU (C239A), Et-OTU (D236A) and Et-OTU (H351A)

Sequence alignment and BLAST analysis results revealed that the protein that interacted with Etv-RDRP was a predicted Et-OTU. The cDNA sequence of the Pp plasmid shared 100% identity with the predicted Et-OTU (GenBank: XM_013374305.1) and 100% identity with the protein sequence*.* Comparative analysis revealed that the C-terminus of the predicted Et-OTU possessed a highly conserved catalytic core containing a Cys box, His box and Asp box (Fig. 3).

### Investigation of et-OTU DUB activity and catalytic core *in vitro*

**Fig. 4:**
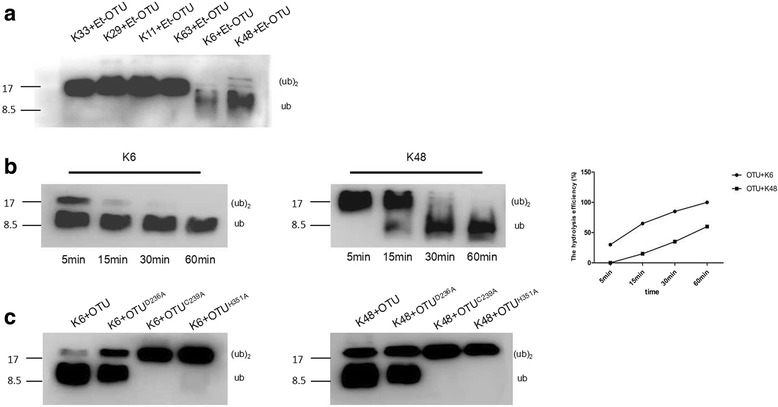
Cleavage assays of purified Et-OTU on di-Ub WT substrates *in vitro*. **a** Purified Et-OTU DUB was incubated with the K48-, K63-, K11-, K29-, K33-, or K6-linked di-Ub chains for 30 min and resolved by Western blotting. **b** Purified Et-OTU DUB was incubated individually with the K48- and K6-linked di-Ub chains for the indicated times. The hydrolytic efficiency of Et-OTU DUB towards K6-linked di-Ub chains was higher than that of Et-OTU DUB towards K48-linked di-Ub chains. **c** Catalytic residues (Cys residue 239, Asp residue 236 and His residue 351) of Et-OTU were individually mutated to Ala. Purified OTUD236A, OTUC239A, OTUH351A and WT OTU DUBs were individually incubated with K48 and K6 di-Ub chains for 30 min

We analysed the substrate specificity of Et-OTU DUB and revealed that it efficiently deconjugated K48- and K6-linked di-Ub chains but not K63-, K11-, K29-, or K33-linked di-Ub chains (Fig. 4a). Therefore, we concluded that Et-OTU exhibits specific hydrolytic activity. We then found that within 30 min, all of K48- and K6-linked Ub dimers were digested into Ub monomers. The activity of Et-OTU DUB towards K6-linked chains was higher than that against K48-linked chains (Fig. 4b). Mutations in the catalytic core (C239A and H351A) eliminated the DUB activity of Et-OTU, whereas the D236A mutation did not (Fig. 4c).

### Combination with Etv-RDRP enhanced et-OTU DUB activity *in vitro*

**Fig. 5:**
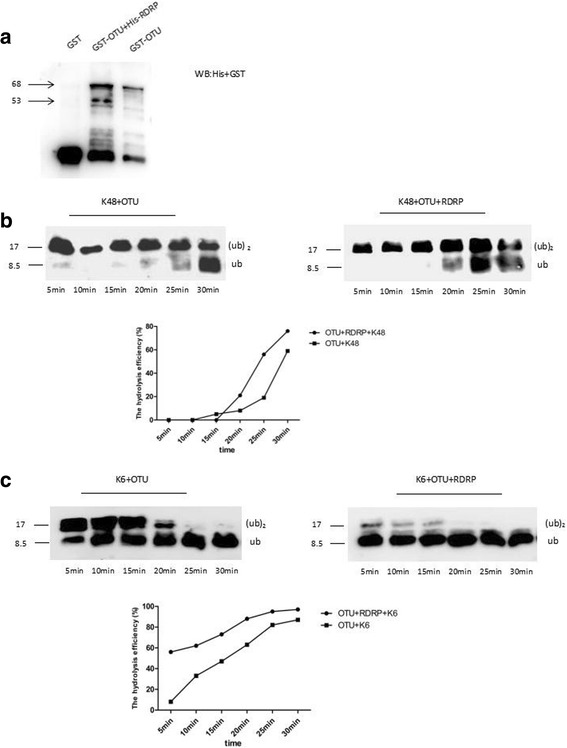
Et-OTU DUB activity was enhanced by Etv-RDRP/Et-OTU complexes in vitro. In vitro deubiquitination assays were performed using Etv-RDRP in combination with Et-OTU and the Et-OTU protein purified from Sf9 as previously described. K48- and K6-linked di-Ub chains were individually used as substrates at a final concentration of 5 μM. The final concentrations of Et-OTU and Etv-RDRP in combination with Et-OTU in the reaction were both 5 μM. **a** Etv-RDRP in combination with Et-OTU and the Et-OTU protein was purified using glutathione-Sepharose 4B and individually analysed by Western blot. **b** In total, 10 μl of both purified Et-OTU DUB and purified RDRP/OTU complexes were separately incubated with 10 μl of K48-linked di-Ub chains for 30 min and resolved by Western blotting. The hydrolytic activity of Et-OTU DUB towards K48-linked di-Ub chains was analysed using ImageJ software. **c** In total, 10 μl of both purified Et-OTU DUB and purified RDRP/OTU complexes were separately incubated with 10 μl of K6-linked di-Ub chains for 30 min and resolved by Western blotting. The hydrolytic activity of Et-OTU DUB towards K6-linked di-Ub chains was analysed using ImageJ software

The GST-OTU protein and GST-OTU-Hexa-tagged-RDRP protein complex were purified using glutathione-Sepharose 4B (Fig. 5a). The hydrolytic efficiency of GST-OTU towards K48-linked chains was lower than that of Etv-RDRP combined with Et-OTU (Fig. 5b). The hydrolytic efficiency of GST-OTU towards K6-linked chains was lower than that of Etv-RDRP combined with Et-OTU (Fig. 5c).

## Discussion

Most protozoan viruses encode RDRP, which consists of a finger, palm and thumb domain. The palm domain, which contains the characteristic Gly-Asp-Asp (GDD) sequence, is the most highly conserved domain in the viral polymerase [21]. Most research on the identification and functions of RDRP-associated proteins has focused on humans, plants and other eukaryotes. However, relationships between virus RDRPs and protozoans have rarely been reported. To examine the relationship between viral RDRP and *E. tenella*, we screened for proteins interacting with Etv-RDRP. The RDRP-interacting protein was revealed to be a predicted Et-OTU by the yeast two-hybrid system, and the OTU-RDRP interaction was confirmed via pull-down, Co-IP and yeast two-hybrid assays. Relationships between OTUs and RDRPs were first described in plants. The turnip yellow mosaic virus (TYMV) ovarian tumour-like protein interacts with the TYMV RDRP, and it can hydrolyse the K48 Ub-conjugated virus RDRP and mediate its stabilisation [22]. Although viral OTUs play important roles in the stability of viral proteins, the regulatory effect of Et-OTU on the stability of viral RDRP needs to be further characterised.

Protein ubiquitination is a versatile posttranslational modification that regulates cellular processes by influencing the stability and function of modified proteins [23, 24]. Single or multiple lysine residues of the protein can be modified by a single Ub protein or Ub oligomers. The Ub chain forms via an isopeptide bond between the C-terminal carboxyl group of Ub and a ε- amino group of lysine residues of another Ub, such as K6, K11, K29, K27, K33, K48 or K63. Importantly, the type of Ub chain determines the functional outcome of the modification [25]. Modification by a K48-linked chain directs modified proteins to the 26S proteasome for subsequent degradation [26]. In yeast, the K48-linked Ub chain also regulates the cell cycle. The K6-linked Ub from parkin can be removed by ubiquitin-specific protease 8 (USP8) to promote parkin turnover, which is required for mitophagy to proceed efficiently [27, 28]. Increasing K6-linked autoubiquitination by silencing USP8 likely occurs to stabilise parkin by protecting it from degradation in the proteasome or lysosome, in line with the role of K6 in stabilising BRCA1/BARD, the other mammalian E3s known to assemble K6-linked Ub chains. BRCA1/BARD1 is implicated in the regulation of numerous cellular processes, including DNA repair, transcription and regulation, as well as cell cycle checkpoint control [29]. Although the functions of K6-linked Ub chains are still unclear, the above data demonstrate the importance of K6-linked Ub chains to radical cellular activities. DUBs can remove ub or poly-Ub chains, and dynamic modification processes constitute a reversible “switch” to regulate and control different substrate protein functions and states, as well as a variety of physiological activities, such as apoptosis, autophagy and cell signalling pathways [30]. Certain diseases, such as cancer, are also associated with the dysfunction of DUBs [31], which are targets for the development of various drugs [32]. Our experiments confirmed that Et-OTU belongs to a DUB family, as it hydrolysed K48- and K6-linked Ub chains *in vitro* but not other Ub chains. The hydrolytic activity of Et-OTU to the K48-linked Ub chain confirms that Et-OTU can affect the progression of the *E. tenella* cell cycle, which is associated with bulk protein turnover. The hydrolytic activity of Et-OTU to the K6-linked Ub chain indicates that Et-OTU may participate in the regulation of mitophagy and mitochondrial integrity and affect the stability and structural integrity of *Eimeria* chromosomal DNA through DNA damage repair.

Telomerase can prevent telomere shortening with cell division and stabilise the end of a chromosome [33, 34]. Some studies have suggested that the cellular abundance of telomerase reverse transcriptase (TERT) protein is regulated through Ub-mediated degradation by several E3 ligases including MKRN1, CHIP, Dyrk2 and HDM2 [35, 36]. Our previous research showed that the Et-OTU protein interacts with *E. tenella* TERT and that down-regulation of Et-OTU can inhibit telomerase activity [19]. This finding indicated that Et-OTU functions through the maintenance of telomerase activity. Although it is unclear whether telomerase is degraded through the modification of K6- or K48-linked Ub chains, the enhancement of Et-OTU activity by Etv-RDRP may contribute to the maintenance of telomerase activity and telomere length by deubiquitination of Et-OTU on telomerase modified by K6- or K48-linked Ub chains. Thus, Etv-RDRP may increase the vitality of *E. tenella* and promote the proliferation of *E. tenella* by enhancing the DUB activity of Et-OTU. The increase in *E. tenella* vitality is helpful for Etv reproduction. The results indicated symbiosis between Etv and *E. tenella* [37]. The enhancement of DUBs by other partners commonly occurs in eukaryotes. For example, USP1, USP12 and USP46 can interact with WD40-repeat protein USP1-associated factor 1 (UAF1), and UAF1 stimulates the catalytic activity of human USP1 through the formation of a tight complex [38]. The USP1/UAF1 complex is required for regulation of the Fanconi anaemia (FA) DNA repair pathway.

The OTU family contains a heterogeneous group of cysteine enzymes. Cysteine proteases show structural similarities in a predicted catalytic domain containing Cys, Asp and His residues, which define the putative catalytic triad of cysteine proteases [39]. Our results confirmed that the loss of DUB activity due to a point mutation in the conserved catalytic core (C239A and H351A) is consistent with the activities of human OTUs, indicating a high level of evolutionary conservation. Although it is conservative, the D236A mutation does not eliminate DUB activity of Et-OTU, indicating that the conserved core (D236) is not Et-OTU active site. The cysteine active site of the *Toxoplasma* DUB TgOTUD3A was identified and is consistent with that of Et-OTU. We analysed the conserved region of TgOTUD3A (GenBank: TGGT1_258780) and found that 57% of Et-OTU and TgOTUD3A amino acids are identical. *Toxoplasma* DUB TgOTUD3A can hydrolyse K48-, K11-, and K63-linked polyubiquitin chains, and modifications by K63-linked chains modulate the functions of modified proteins [40]. Lys11-linked chains constitute an alternative degradation signal used during cell cycle progression [41], showing that TgOTUD3A is associated with cell cycle-related functions. TgOTUD3A knockout (TgOTUD3A-KO) parasites exhibit a complex phenotype associated with the fidelity of parasite replication. However, the effect of Et-OTU knockout parasites on the biological characteristics of *E. tenella* remains unknown.

We identified RDRP-associated proteins using a yeast two-hybrid system with the pGBKT7-RDRP plasmid from an *E. tenella* cDNA library as bait and obtained the full-length cDNA of the predicted Et-OTU, confirming the interaction between Et-OTU and Etv-RDRP. The deubiquitination active site and specificity of Et-OTU were also analysed *in vitro*. Furthermore, combining Etv-RDRP with Et-OTU enhanced the DUB activity of Et-OTU *in vitro*. This research provided insights into the symbiosis between *E. tenella* and Etv.

## Conclusions

The predicted Et-OTU interacts with RDRP and efficiently deconjugates the K48- and K6-linked di-Ub chains *in vitro*. Etv-RDRP enhances the DUB activity of Et-OTU *in vitro*. The results of this study indicated that the combination of Etv-RDRP and Et-OTU participates in the progression of the *E. tenella* cell cycle and may also affect the proliferation of *E. tenella*. This discovery is a promising start for further studies on the effect of Etv on the molecular biological characteristics of *E. tenella.*

## Abbreviations

DUBs: Deubiquitinases
GDV: *Giardia duodenalis* virus
LRV: *Leishmania* RNA virus
OTU: Ovarian tumour
Ppplasmid: Positive prey plasmid
RDRP: RNA-dependent RNA polymerase
TYMV: Turnip yellow mosaic virus
USP: Ubiquitin-specific protease
